# Is Thymidine Glycol Containing DNA a Substrate of *E. coli* DNA Mismatch Repair System?

**DOI:** 10.1371/journal.pone.0104963

**Published:** 2014-08-18

**Authors:** Svetlana A. Perevozchikova, Roman M. Trikin, Roger J. Heinze, Elena A. Romanova, Tatiana S. Oretskaya, Peter Friedhoff, Elena A. Kubareva

**Affiliations:** 1 Department of Chemistry and Belozersky Institute of Physico-Chemical Biology, Lomonosov Moscow State University, Moscow, Russia; 2 Institute of Cell Biology, University of Bern, Bern, Switzerland; 3 Institute for Biochemistry, Justus Liebig University, Giessen, Germany; Institute of Molecular Genetics IMG-CNR, Italy

## Abstract

The DNA mismatch repair (MMR) system plays a crucial role in the prevention of replication errors and in the correction of some oxidative damages of DNA bases. In the present work the most abundant oxidized pyrimidine lesion, 5,6-dihydro-5,6-dihydroxythymidine (thymidine glycol, Tg) was tested for being recognized and processed by the E. coli MMR system, namely complex of MutS, MutL and MutH proteins. In a partially reconstituted MMR system with MutS-MutL-MutH proteins, G/Tg and A/Tg containing plasmids failed to provoke the incision of DNA. Tg residue in the 30-mer DNA duplex destabilized double helix due to stacking disruption with neighboring bases. However, such local structural changes are not important for *E. coli* MMR system to recognize this lesion. A lack of repair of Tg containing DNA could be due to a failure of MutS (a first acting protein of MMR system) to interact with modified DNA in a proper way. It was shown that Tg in DNA does not affect on ATPase activity of MutS. On the other hand, MutS binding affinities to DNA containing Tg in G/Tg and A/Tg pairs are lower than to DNA with a G/T mismatch and similar to canonical DNA. Peculiarities of MutS interaction with DNA was monitored by Förster resonance energy transfer (FRET) and fluorescence anisotropy. Binding of MutS to Tg containing DNAs did not result in the formation of characteristic DNA kink. Nevertheless, MutS homodimer orientation on Tg-DNA is similar to that in the case of G/T-DNA. In contrast to G/T-DNA, neither G/Tg- nor A/Tg-DNA was able to stimulate ADP release from MutS better than canonical DNA. Thus, Tg residue in DNA is unlikely to be recognized or processed by the *E.* coli MMR system. Probably, the MutS transformation to active “sliding clamp” conformation on Tg-DNA is problematic.

## Introduction

DNA in cells is continuously exposed to various genotoxic agents, such as chemically active compounds, ultraviolet and ionizing radiation. In many cases, this exposure leads to formation of reactive oxygen species which are capable of attacking DNA at sugar-phosphate backbone or heterocycles. The lesions formed may eventually result in cell transformations and a variety of diseases including cancer [1]. Oxidation of the double bond of thymidine gives rise to 5,6-dihydro-5,6-dihydroxythymidine (thymidine glycol, Tg) which is the most abundant oxidized pyrimidine nucleotide [2], [3]. Approximately 400 Tg residues are formed in one cell per day [4]. Tg contains two chiral centers (at C5 and C6 atoms). Thus, four Tg stereoisomers exist: *cis*-(5*R*,6*S*), *trans*-(5*R*,6*R*), *cis*-(5*S*,6*R*), *trans*-(5*S*,6*S*). The *cis*-forms are predominant in nature [5]. Repair of Tg is carried out by endonucleases: EndoIII and EndoVIII in *E. coli* and by their eukaryotic homologues, hNth1 and hNeil1 [6]. Also, several glycosylases, for instance human thymine-DNA glycosylase (TDG) and m^5^CpG binding protein MBD4, remove Tg from G/Tg pair [7].

Tg residue is most common in A/Tg and G/Tg pairs. In the first case thymidine is oxidized in a Watson-Crick A/T pair. Nonetheless, thymidine is oxidized more frequently as a partner of G in G/T pair, which is a common result of replication error or deamination of 5-methyl-2′-deoxycytidine [8]. G/T pair takes a wobble conformation, which makes the double bond between C5 and C6 atoms of thymidine more vulnerable to oxidizing agents [9], [10]. G/T pair in DNA are most effectively corrected by DNA mismatch repair (MMR) system [11]. It has been mentioned in the past that the MMR system is important in the processing of Tg containing DNA (Tg-DNA) [12], [13], however there has been no direct experimental evidence for that conclusion so far. It is well documented that some DNA repair pathways have overlapping specificities, giving rise to the need to coordinate their activities and repair different substrates [14], [15]. For example, the MMR system is involved in processing of certain chemical modifications in DNA, e.g. O6-methylguanosine [16], [17], 8-oxoguanosine [18], [19], adducts formed during exposure of carcinogens on DNA [20], photo-induced products [21]–[23], and products of the reaction of DNA with cisplatin derivatives [24]. Here we addressed the question whether thymidine glycol is recognized and processed by the MMR system in *E. coli.*

A general scheme of mismatch repair in E. coli is presented in [25]. The first and principal repair protein in this pathway is MutS, which recognizes the mismatch. The MutS-DNA complex then recruits a second protein, MutL, which in turn activates the latent endonuclease MutH. MutH hydrolyses the daughter DNA strand which is temporarily non-metylated at 5′-GATC-3′ sites. DNA helicase binds to DNA at position of single-stranded break and unwinds DNA. Then a non-methylated DNA strand is hydrolyzed by a set of exonucleases. The single-strand binding protein (SSB) protects a methylated (parental) DNA strand. The resulting gap in the DNA is rebuilt by DNA polymerase III. DNA ligase restores the integrity of the corrected strand [25].

**Figure 1:**
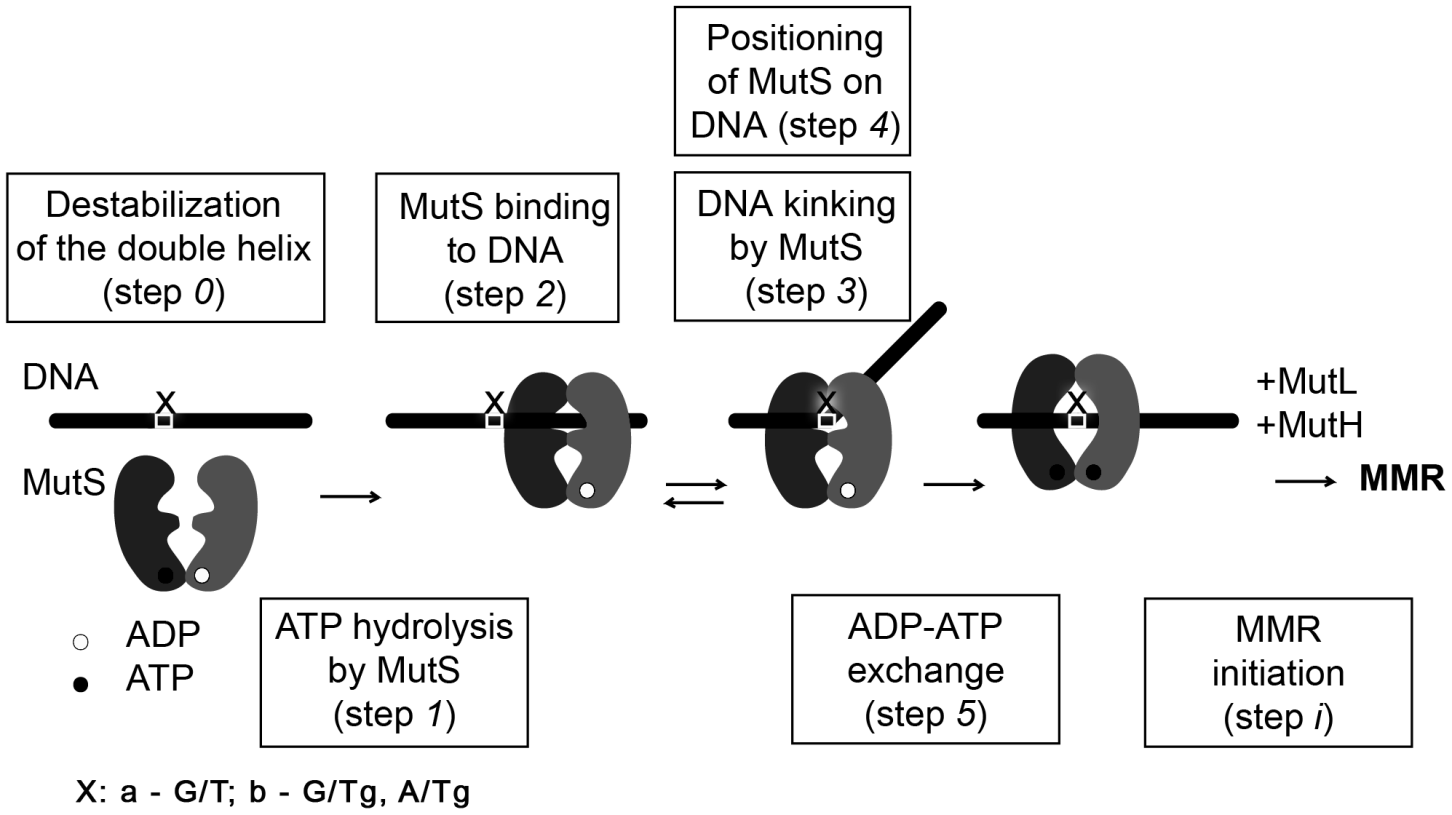
The general scheme of MutS homodimer interaction with DNA. The issues investigated in the current work are enclosed in the rectangles.

According to [26], [27] MutS from *E. coli* is a homodimeric ATPase, which forms an integrated DNA binding site, embracing DNA in a clamp-like structure (Figure 1). MutS functions as a sensor of local destabilization of DNA, caused by appearance of a non-complementary pair of nucleotides (Figure 1, step *0*, **a**). The two main functions of MutS – DNA and ATP binding – are interrelated, but the subunits are not identical with respect to DNA binding and ATP hydrolysis. To locate mismatches in DNA, MutS scans it in a process of linear diffusion [28]. Based on the results of [29] at this moment hydrolysis of ATP to ADP in one of subunits of MutS may take place (Figure 1, step *1*). Then MutS forms specific contacts with the mismatch in a so called initial recognition complex [10], [30]. This complex is characterized by a DNA bending at 60° (Figure 1, steps *2–4*, **a**). Then an exchange of ADP with ATP in ATP domains of MutS takes place (Figure 1, step *5*, **a**) and there is formed an ultimate recognition complex, which being in the “sliding clamp” conformation initiates an MMR reaction cascade involving MutL и MutH (Figure 1, step *i*, **a**).

The starting point of our investigation was to answer the question whether the presence of Tg (5R, 6S-stereoisomer) influences the mismatch-provoked incision of DNA by MutS, MutL and MutH (Figure 1, step *i*, **b**). If it does, it would be necessary to study the further steps of MMR processing of Tg-DNA, e.g. connecting with UvrD, exonucleases, polymerase III and ligase functioning. If it does not, we have to find the reason of this phenomenon and to study the earliest MMR stage - MutS interaction with G/Tg and A/Tg containing DNAs in comparison with canonical and G/T bearing duplexes. In this case we planned to test the Tg influence on DNA stability (Figure 1, step *0*, **b**), the DNA-stimulated ATP hydrolysis by MutS (Figure 1, step *1*), the affinity of MutS to DNA duplexes (Figure 1, step *2*), the kinking of DNA in the complex with MutS (Figure 1, step *3*), the positioning of MutS on the DNA (Figure 1, step *4*), the rate of nucleotide exchange in ATPase domain (Figure 1, step *5*).

## Materials and Methods

### Recombinant Proteins

The recombinant MutS, MutL and MutH proteins were expressed in *E. coli* strains and purified using Ni-NTA affinity chromatography followed by gel filtration as described in [31], [32]. The total concentrations of the proteins were estimated spectrophotometrically at 280 nm. The active MutS concentration was determined by the Scatchard approach [33].

### Synthetic DNA Fragments

The oligonucleotides containing the thymidine glycol residue (5R, 6S-stereoisomer) in a specified position were prepared as described in [34]. The oligodeoxyribonucleotides (unmodified as well as containing Alexa-488 or Alexa-594 dyes) were provided by Eurogentec (Belgium). Oligonucleotide duplexes I–IV were annealed from the corresponding oligonucleotides in 10 mM HEPES buffer (pH 7.5) with 125 mM KCl.

The structures of the duplexes I–IV (5′→3′/3′→5′) are shown below.

CAAGCCTATGCCCTCAGCACCCA**G**GGTGCCI (G/C)

GTTCGGATACGGGAGTCGTGGGT**C**CCACGG

CAAGCCTATGCCCTCAGCACCCA**G**GGTGCCII (G/T)

GTTCGGATACGGGAGTCGTGGGT**T**CCACGG

CAAGCCTATGCCCTCAGCACCCA**G-**GGTGCCIII (G/Tg)

GTTCGGATACGGGAGTCGTGGGT**Tg**CCACGG

CAAGCCTATGCCCTCAGCACCCA**A-**GGTGCCIV (A/Tg)

GTTCGGATACGGGAGTCGTGGGT**Tg**CCACGG

**Figure 2:**
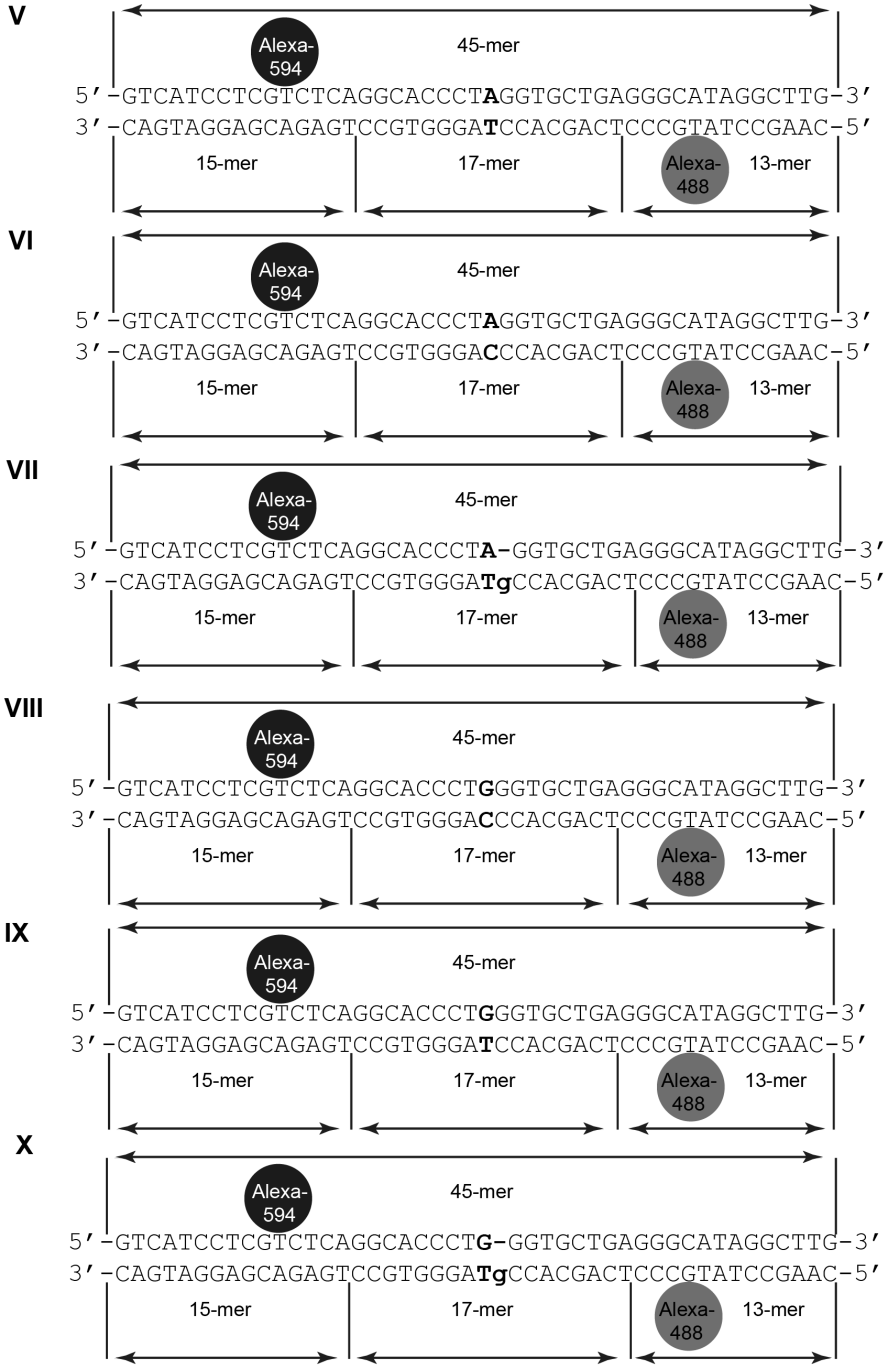
45 bp duplexes V-X containing the variable nucleotide pair and the FRET pair. Variable nucleotide pair is shown in bold. Alexa-594 (black circle) and Alexa-488 (grey circle) are linked to T residues.The duplexes are obtained by hybridization of three fragments (15-, 17- and 13-mer) on the 45-mer template strand. The nicks in the “bottom” strand of duplexes are indicated by vertical lines.

The DNA systems V-X under investigation are 45 bp duplexes with the central variable nucleotide pair and two fluorophores (Figure 2). The design of duplexes V-X contained Alexa-488 (fluorescence donor) in one strand and Alexa-594 (fluorescence acceptor) in the other one was performed as in [35]. The 45-mer oligonucleotide carrying Alexa-594 served as a template and was hybridized with modified (carrying Alexa-488 or Tg) or unmodified 15- or 17-mer oligonucleotides. The excess of the short oligonucleotides relative to the 45-mer template did not exceed 10% in all reaction mixtures. To stabilize the duplexes, the hybridization buffer contained 5 mM MgCl_2_. The formation of the fluorescently labeled duplexes was controlled by electrophoretic mobility shift assay (EMSA) in 20% polyacrylamide gel (PAG) (Figure S1 in File S1). The formed DNA duplexes were stored at 4°C and used within 2 weeks.

### Plasmid DNA Preparation and Testing of Mismatch-Provoked MutH Endonuclease Activity

The initial plasmid pUC-MMR was multiplied in XL1 blue *E. coli* strain (Stratagene, USA) and purified using the Wizard *Plus* SV DNA purification system (Promega, USA). The generation of 3315 bp covalently closed circular DNA (cccDNA) containing a single mismatch or a thymidine glycol residue at position 169 and a single hemimethylated 5′-GATC-3′/5′-Gm^6^ATC-3′-site at position 356 was performed as in [36] (Figure S2 in File S1). The incision of the obtained plasmids by MutS-MutL-MutH complex was tested as described in [36]. The reaction mixture of cccDNA (25 nM) was incubated with MutS (200 nM), MutL (200 nM), and MutH (50 nM) (total concentrations of proteins per monomer). The reactions were performed in buffer containing 150 mM KCl and 1 mM ATP at 37°C. Incubation time varied from 0 to 10 min. The reaction mixtures were analyzed in 1% agarose gel containing ethidium bromide as a reagent for DNA visualization. The experiments were repeated 5 times, and the mean standard deviation (SD) was calculated (Figure S3 in File S1).

### Thermal Stability of DNA Duplexes

UV absorption of DNA duplexes I–IV (0.6–1.0 µM) *vs* temperature was measured in a 300 µl quartz microcuvette (Hellma, Germany) with a 10 mm path length using the U-2900 UV/Visible Spectrophotometer (Hitachi, Japan) equipped with a thermoelectric temperature controller. The thermally induced unfolding transitions were monitored between 25 and 85°C at 260 nm with the heating rate of 0.5°C/min. Melting curves were obtained in integral form (at least 3 times for each duplex) and processed using GraphPad Prism 5 (GraphPad Software, Inc., USA). Hypochromic effect was calculated by the equation: 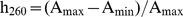. The duplex melting temperature (T_melt_) and the ΔT values, which characterize the DNA melting cooperativity, were determined from the differential melting curves (Table 1). The mean SD was calculated.

**Table 1:** Characteristics of DNA duplex thermal stabilities.

Duplex number (X/Y)^a^	T_melt_, °C (±1)^b^	h_260_, % (±1)^c^	C_D_, 10^−6^ M^d^	ΔT,°C (±1)^e^
I (G/C)	79	24	0.84	16
II (G/T)	74	23	0.98	18
III (G/Tg)	63	21	0.70	20
IV (A/Tg)	67	21	0.62	19

a Variable nucleotide pair.

b Melting temperature of duplex.

c Hyperchromic effect.

d Concentration per duplex.

e Cooperativity of phase transition.

The averaged data of three experiments are presented. The error of the mean indicates a SD.

### Radioactive Labeling of DNA Duplexes

The label was introduced at 5′-end of oligonucleotides (30–40 pmol) using [γ-^32^P]ATP (0.3 µM) and T4 polynucleotide kinase (Thermo Fisher Scientific, USA). The labeled oligonucleotides were purified by electrophoresis in a 20% PAG with 7 M urea followed by elution from the gel.

### Steady-State ATPase Activity of MutS

The reaction mixture (50 µl) contained 20 mM HEPES buffer (pH 8.0), 125 mM KCl, 5 mM MgCl_2_, 1 mg/ml BSA, 200 nM DNA duplex (or without DNA duplex) and 200 nM of MutS (per monomer). The mixture was incubated for 5 min at 25°C. Reactions were initiated by adding 2 µM ATP, including [γ-^32^P]ATP. Aliquots of 5 µl were collected with 1 min intervals during 10 min and mixed with 5 µl of STOP-buffer (0.5 M EDTA: formamide  = 4: 1). Each experiment was performed at least 3 times. The products of [γ-^32^P]ATP hydrolysis were analyzed by electrophoresis (20 V/cm) in a 30% PAG with 7 M urea (Figure S3A in File S1). The radioactive bands in gels were visualized by autoradiography using Fujifilm FLA-3000 scanner (USA). The proportion of radioactive phosphate was calculated by AIDA 3.44.035 software and the data were plotted (Figure S3B in File S1). The slope of the linear part of the curve was considered as the rate of ATP hydrolysis by MutS and was summarized in Table 2 (first column). The standard error of the mean (SEM) was calculated.

**Table 2:** Parameters of MutS binding to DNA, ATP hydrolysis and nucleotide exchange in ATPase domain of protein.

Duplex number (X/Y)^a^	Initial rates (*v*_0_) of ATP hydrolysis by MutS		Dissociation constants (*K*_d_) of MutS complexes with DNA	Rate constants of ADP exchange (*k*_off_^mant-ADP^) in ATPase domain of MutS	
	*v*_0_, nM·s^−1^	Rel. *v*_0_ b	*K*_d_, nM	*k*_off_, s^−1^	Rel. *k*_off_ c
MutS without DNA	0.37±0.018	1.0	-	0.012±0.001	1.0
I (G/C)	0.77±0.069	2.1	32±3.3	0.047±0.001	3.9
II (G/T)	0.75±0.073	2.0	7±1.5	0.28±0.013	23.0
III (G/Tg)	0.66±0.036	1.8	44±2.3	0.037±0.015	3.0
IV (A/Tg)	0.69±0.011	1.9	25±3.0	0.025±0.001	2.1

a Variable nucleotide pair.

b The ratio of *v*_0_ of ATP hydrolysis by MutS in the absence or in the presence of duplexes I–IV to *v*_0_ of ATP hydrolysis by MutS in the absence of DNA.

c The ratio of rate constant of mant-ADP dissociation from its complex with MutS (*k*_off_^mant-ADP^) in the absence or in the presence of duplexes I–IV to *k*_off_^mant-ADP^ in the absence of DNA.

The error of the mean indicates a SEM.

### MutS Binding to DNA

MutS binding to DNA duplexes was characterized by the Scatchard assay [33] according to the reaction scheme 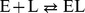. The total MutS concentration per monomer was kept constant (90 nM), while the concentrations of the ^32^P-labeled DNA duplexes varied from 1 to 20 nM. The binding was carried out at 37°C for 20 min in 20 µl of the following buffer: 20 mM HEPES (pH 8.0), 125 mM KCl, 1 mM ADP, 5 mM MgCl_2_, 1 mg/ml BSA, 9% (v/v) glycerol. Each experiment was performed at least 3 times. The formation of the protein–DNA complexes was analyzed by EMSA in non-denaturing 4% PAG (18×22×0.15 cm; TAE-buffer, 4.5 V/cm) for 90 min at 4°C (Figure S4A in File S1). The gel was dried in vacuum under heat. The radioactive bands were visualized by autoradiography using Fujifilm FLA-3000 scanner (USA). The intensities of the radioactive bands corresponding to the complex (MutS•DNA) and the free DNA were measured. The fraction of the DNA–protein complex was calculated as the ratio of its intensity to the total intensity in the lane; the percentage was converted to the molar concentrations considering that the total DNA amount in the reaction mixture corresponded to the 100% intensity in the lane. The [MutS•DNA]/[DNA] values were plotted versus [MutS•DNA] and approximated by linear function (Figure S4B in File S1). The dissociation constant (*K*_d_) was calculated from the slope of the line (Table 2, second column). The error of the mean indicates a SEM.

### Steady-State Fluorescence Measurements

MutS (total concentration per monomer 0–400 nM) was mixed with DNA duplexes V–X (20 nM) in 100 µl of 25 mM Tris-HCl buffer (pH 7.5) containing 125 mM KCl, 5 mM MgCl_2_, and 1 mM ADP and kept for 5 min at 20°C. Fluorescence intensity (*I*) and anisotropy (*r*) were measured using Tecan Infinite F200 fluorescence plate reader (Switzerland). Three filter sets were used, namely Green (λ_ex_ = 475 nm, λ_em_ = 525 nm), Red (λ_ex_ = 575 nm, λ_em_ = 625 nm), and FRET (λ_ex_ = 475 nm, λ_em_ = 625 nm) [35]. The anisotropy values for the maximal binding of MutS with the duplexes V–X were estimated (Figure S5 in File S1). Change in the efficiency of energy transfer was calculated. Each experiment was performed in 3 timed, and the SD was determined (Table 3).

**Table 3:** Change in energy transfer efficiency upon MutS binding to different DNA duplexes.

Duplex number (X/Y)^a^	Δ(F_max_/Red)
V (A/T)	0.01±0.001
VI (A/C)	0.02±0.002
VII (A/Tg)	0.01±0.008
VIII (G/C)	0.01±0.001
IX (G/T)	0.13±0.001
X (G/Tg)	0.01±0.001

a Variable nucleotide pair.

The error of the mean indicates a SEM.

### Kinetics of nucleotide exchange in the ATPase domain of MutS

The initial mixture contained 25 mM HEPES (pH 7.5), 125 mM KCl, 5 mM MgCl_2_, 0.05% (*v/v*) TWEEN-20, and 500 nM 2′-(or-3′)-O-(N-methylanthraniloyl)adenosine-5′-diphosphate (mant-ADP) in 75 µl. The investigations were done in the presence or absence of duplexes I–IV (200 nM). The measurements were performed at 25°C using spectrofluorometer Varian Cary Eclipse (USA). The fluorescence intensity was recorded over time under the following conditions: λ_ex_ = 355 nm, λ_em_ = 448 nm, 600 V at the lamp, slit widths of 20 nm. The initial part of the experimental curve was recorded in the absence of the protein. Then MutS was added into the cuvette up to the total concentration per monomer of 800 nM. The solution was mixed by quick pipetting and the measurement continued. When the signal reached plateau and stabilized, ADP was quickly added to the cuvette up to the concentration of 1 mM. The mixture was stirred and the signal recording was continued until reaching another plateau (Figure S6 in File S1).The drop of fluorescence was estimated. The data were processed using the approach reported in [37], [38]. The single exponential decay equation was used for fitting the experimental curve in the GraphPad Prism 4 program (GraphPad Software, Inc., USA). Thus, the dissociation rate constants of the MutS complex with mant-ADP (*k*_off_^mant-ADP^) were calculated. The measurements were done 3–5 times. The error of the mean indicates a SEM (Table 2, last column).

## Results and Discussion

### Does Tg influence the mismatch-provoked incision of DNA by MutS, MutL and MutH?

**Figure 3:**
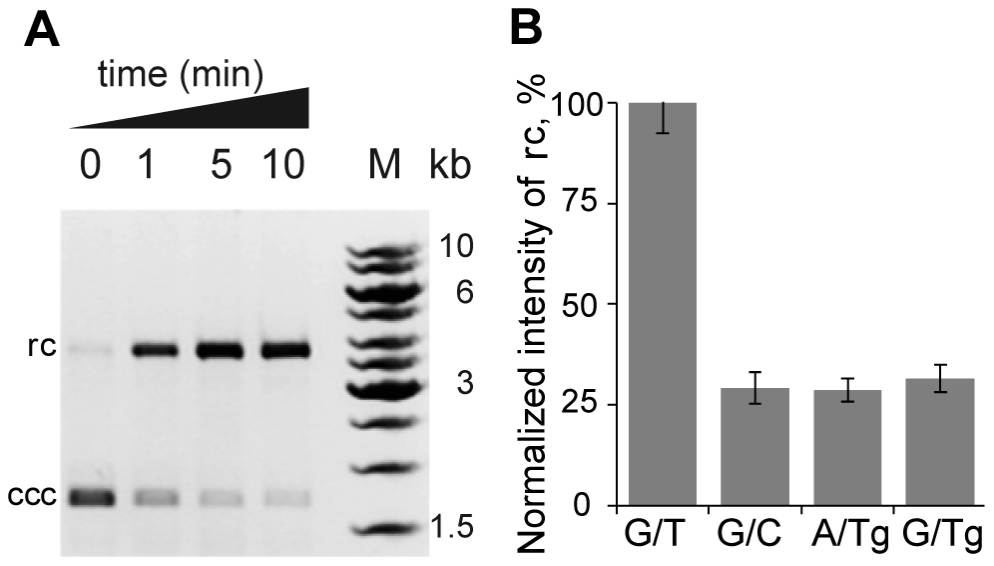
The plasmid DNAs cleavage by MutH in a MutS-MutL dependent manner. **A**, Analysis of the G/T-cccDNA treated with MutS-MutL-MutH mixture after 1, 5 or 10 min incubation in 1% agarose gel containing ethidium bromide. The initial cccDNA is shown (0 min). M – DNA ladder. **B**, Diagram representing the data of hydrolysis by MutS-MutL-MutH mixture of G/T-, G/C-, G/Tg- and A/Tg-cccDNA (the variable nucleotide pair introduced in cccDNA is indicated under the lanes) for 5 min. The experiments were performed 5 times. Error bars are standard deviations of the mean.

The most decisive answer to the question about the possibility of a damage repair by *E. coli* MMR system in vitro experiments can be obtained by estimating the MutH endonuclease activity in the presence of proteins – MutS and MutL (Figure 1, step *i*, **b**). The purified proteins MutS, MutL and MutH, as well as specially constructed DNA, which contains the damaged fragments and the recognition site of MutH were used in these experiments. To investigate the influence of the Tg on DNA incision by complex of MutS-MutL-MutH proteins two Tg containing plasmid DNAs, with A/Tg and G/Tg pairs, as well as the positive control (with G/T mismatch) and the negative control (with G/C pair) have been obtained (Figure S2 in File S1). The covalently closed circular (ccc) DNA is nicked during the incubation with MutS-MutL-MutH mixture and relaxed circular (rc) plasmid DNA accumulates (Figure 3A). The increase of the fluorescence intensity of the rc DNA band has been quantified. In our experiments the G/T-cccDNA was incised almost completely within 5 min (Figure 3A). The results for G/T-, G/C-, G/Tg- and A/Tg-cccDNA cleavage by MutS-MutL-MutH mixture are presented in Figure 3B. The G/C-cccDNA was hydrolysed 4 times less effectively than G/T-cccDNA under the same conditions. There is no change over time in this activity level observed with the G/C- and G/Tg-cccDNA (the data are not shown). DNAs containing either G/Tg or A/Tg were not incised significantly better than the G/C-cccDNA. It is possible to suppose that no one of the Tg containing DNAs are able to initiate MMR.

The lack of repair initiation of Tg containing DNA could be due to peculiarities of the local structure of the nucleotide pair with an oxidized base and as a result a failure of MutS to interact with modified DNA at one of several steps during MMR initiation. According to the existing data, both MutS and DNA undergo significant conformational changes while interacting [39], [40]. Moreover, nucleotide exchange and ATP hydrolysis in ATPase domains of MutS are coordinated with conformational changes required for protein-protein interactions with MutL and DNA-protein interactions [25], [41]. Thus, it is necessary to determine the influence of Tg on DNA structure in our experimental conditions and whether one of the steps of MutS interaction with A/Tg and G/Tg containing DNAs is crucial for DNA repair (Figure 1).

### The Tg influence on DNA stability

Local instability of DNA double helix can serve as a signal to activate a number of repair systems [42]. That is why, there was clarified the destabilizing effect of thymidine glycol residue in G/Tg and A/Tg pairs in DNA (Figure 1, step *0*, **b**; Table 1).The 30 bp DNA I–IV were used to estimate the influence of Tg incorporation on duplex stability. The method of UV spectroscopy was applied for investigation. The results were evaluated by the changes in melting temperatures of duplexes (T_melt_). Previously it was shown that 30 bp G/T-duplex forms sufficiently stable complexes with MutS and can be used in further investigations [10].

Thermodynamic properties of duplexes I–IV are presented in Table 1. Wobble G/T pair in DNA duplex II locally destabilizes the structure of the double helix, resulting in a decrease of 5°C in T_melt_ in comparison with duplex I. As expected, the DNAs containing G/Tg (III) and A/Tg (IV) pairs are even less stable than G/T duplex II. Their T_melt_ decreased by 16 and 12°C, respectively, in comparison with canonical duplex I. These results are consistent with the published data [9], [43] obtained for duplexes of different lengths or sequences and in the other buffer conditions.

The presence of G/T pair does not affect the hypochromia of complex formation of 30 bp DNA duplex, while the lower values of h_260_ are typical for DNA duplexes III and IV, containing Tg residue (Table 1). For the canonical DNA duplex I helix-coil transition occurs in the range of 16°C. Introduction of a non-complementary G/T pair in duplex II reduces the melting cooperativity of DNA by 2°C. This effect is somewhat stronger for Tg containing duplexes III and IV (by 4 and 3°C, respectively). All the data combined indicate a local destabilization of the DNA duplex structure in the A/Tg and G/Tg pairs.

The reason for the destabilizing effect of thymidine glycol most likely lies in the loss of aromaticity in a heterocyclic base and a derangement of the interplanar interactions [2]. Nevertheless, the lack of initiation of the repair of plasmid DNA containing the Tg by MutS-MutL-MutH mixture (see above, Figure 3B) demonstrates that local destabilization of the double helix itself is a necessary but not a sufficient factor in recognizing a defect in DNA by MMR system.

### The Steady-State ATP Hydrolysis in the ATPase Domains of MutS

MutS is a weak ATPase belonging to the ABC-family. The ATP hydrolysis is necessary for the MutS transition from one conformational state to another (Figure 1, step *1*, **a**). The events occurring in the DNA binding center and those in the ATPase domains are interconnected. Two different models are proposed for the ATP hydrolysis in the ATPase domains of MutS. According to the first one, MutS switches from the “turned-off” state (incapable of DNA binding) to the “turned-on” state (which is able to bind DNA) after the ATP hydrolysis [44]. According to the other model [29], the ATP hydrolysis takes place continuously while MutS is scanning the mismatched DNA. In the latter case, the DNA structure could influence the ATPase activity of the protein. However, no clear understanding of the coordination between the ATPase domains and the function of the whole MutS molecule has been developed to date. In the present work, we studied the influence of thymidine glycol presence in DNA onto the MutS ATPase domain activity (Figure 1, step *1*, **b**).

The MutS ATPase activity in the absence of DNA and in the presence of duplexes I–IV is evaluated by monitoring of the hydrolysis of radioactively labeled ATP. [γ-^32^P]ATP is cleaved by MutS forming non-radioactive ADP and ^32^P-containing phosphate (Figure S3A in File S1). Under the conditions used, the hydrolysis represents a classic steady-state kinetics [45] (Figure S3B in File S1). The initial rates (*v*_0_) of the ATP hydrolysis by MutS under different conditions and their values relative to *v*_0_ (Rel. *v*_0_) of the ATP hydrolysis in the absence of DNA are presented in Table 2 (first column). In the presence of DNA, *v*_0_ of the ATP hydrolysis is approximately 2 times higher than the initial rates observed in the absence of DNAs. This result is in accordance with the published data [37] and testifies the interconnection between the DNA binding and the ATP hydrolysis by MutS. Nevertheless, the initial rate of the ATP hydrolysis by MutS remains in the same range in the presence of any of the duplexes under our experimental conditions. Duplex II containing a G/T pair is the preferable ligand for MutS [46] (see also below). However, it accelerates the reaction to the same extent as duplex I containing a canonical G/C pair, although this DNA demonstrated low affinity to MutS [46]. Similar *v*_0_ values of the ATP hydrolysis were obtained in the presence of Tg containing duplexes III and IV. Thus, the Tg residue in DNA has no influence on the MutS capability to hydrolyze ATP.

### MutS Binding to the Thymidine Glycol Containing DNA

The next step was the estimation of MutS affinity to Tg-DNA in the presence of ADP (Figure 2, step *2*). Oxidized thymidine can prevent formation of specific protein-DNA complexes. The dissociation constants for MutS complexes with DNAs I–V were determined by the Scatchard approach [33]. MutS formed a single complex with each one of duplexes I–IV (Figure S4 in File S1). The values of dissociation constants (*K*_d_) of protein-DNA complexes are presented in Table 2 (second column).

MutS binds the duplex II containing the G/T pair with the highest affinity (*K*_d_∼7 nM). As expected, MutS binds the canonical DNA duplex I with a smaller affinity (*K*d ∼32 nM), since it is not a substrate for the mismatch repair system. The most stable MutS complex with DNA is formed in the case of unpaired thymidine; the second in the row is the DNA containing a G/T pair, and the weakest complex is observed with the canonical DNA [46]. The *K*_d_ value for the DNA duplex III containing a G/Tg pair is even higher (*K*_d_∼44 nM) than that for the canonical duplex I. Thus, G/Tg duplex should be “a bad substrate” for MMR. The most important feature in the MutS complex with mismatch containing DNA is the interaction between Phe36 (numbering as in *E. coli*) and the mismatched thymine. Thymine glycol is not aromatic and therefore non-planar which makes impossible its stacking with Phe36 (Figure 1, steps *2* and *3*, **a**).

Moreover, the methyl group at C5 atom of thymine was shown to interact with MutS when it formed the specific complex with the G/T containing DNA. While the methyl group of thymine lies in plane of the heterocycle, that of thymine glycol may be either equatorial or axial [2], [47] and therefore could be a steric obstacle for the MutS interaction with DNA. These factors may lead to a significant decrease of MutS affinity to the G/Tg-DNA in comparison with the G/T-DNA. As published previously, the complex of MutS with DNA containing an abasic site opposite T has a lower *K*_d_ value compared to the complex with G/T containing DNA. Conversely, the *K*_d_ value for the MutS complex with DNA containing the abasic site opposite G is 4-fold higher [46]. One can conclude that the aromaticity loss of T affects MutS interaction with DNA in the same way as the abasic site opposite G does.

The *K*_d_ for MutS complex with A/Tg-duplex IV (∼25 nM) is lower than that of duplex I but three times higher than that of G/T containing duplex II.

Interestingly, the MutS affinity to the “imperfect” duplexes with G/T, A/Tg, and G/Tg pairs correlates with the thermal stability of these double stranded DNAs (Table 1): G/T-DNA is the most stable (74°C), A/Tg-DNA is less stable (67°C), and G/Tg-DNA is the least stable (63°C). The tendency, that a duplex containing the more destabilizing mismatch forms the weaker complex with MutS, has been previously observed [40], [48]. Thus, the G/T mismatch does not disturb DNA structure significantly and is recognized better than other mismatches, whereas C/C considerably destabilizes the double helix and is almost ignored by MutS and not repaired by MMR.

### Estimation of DNA Kink during Binding with MutS

DNA bending is crucial for MMR repair systems, as shown by X-ray crystallography [10], [30]. DNA is kinked (60°) at the mismatch in the specific complex with MutS (Figure 1, step *3*, **a**). The kinking of the DNA duplexes in the complex with MutS was monitored by FRET as described before [36], [49]. The 45 bp duplexes with a FRET pair were designed (Figure 2). Fluorophores Alexa-488 (donor) and Alexa-594 (acceptor) were put in the DNA at the distance of 25 bp which is close to the Förster radius of this FRET pair. As a result of DNA bending by protein, the distance between the fluorophores attached to the DNA becomes shorter and the efficiency of energy transfer is enhanced. Duplexes V-X differ from each other by the central nucleotide pair: mismatch (G/T, A/C), canonical (A/T, G/C) or with Tg residue (G/Tg, A/Tg). The presence of nicks in one of the DNA strands did not affect the binding of MutS with duplexes [35].

**Figure 4:**
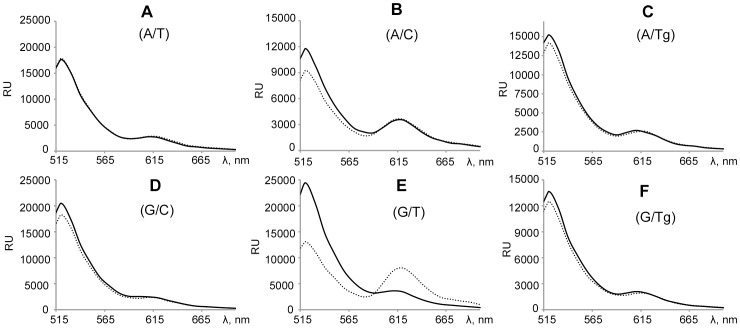
Fluorescence emission spectra. Panels **A–F** correspond to DNA duplexes V–X in the presence of MutS (400 nM per monomer – dashed line) or in the absence of protein (solid line). DNA duplexes (concentration 20 nM) contain FRET pair - Alexa-488 (donor) and Alexa-594 (acceptor). The central variable nucleotide pair in DNA is shown in parentheses. The samples were irradiated by light at 470 nm. Spectra were recorded at 500-800 nm. RU - the signal detector in stated units. Each spectrum was recorded at least three times. The figure shows one of the experiments.

To estimate the Tg influence on DNA kinking (Figure 1, step *3*, **b**), FRET during MutS binding with duplexes V-X was measured. Fluorescence spectra were recorded for solutions containing either free or MutS bound DNA (Figure 4). The most efficient energy transfer occurred for G/T-DNA, where fluorescence extinguishing happened in the case of the donor, and the intensity doubled for the acceptor. For A/C-DNA, only inconsiderable change in FRET was observed. The spectra for other duplexes in complexes with MutS underwent a negligible change. The change of the efficiency of energy transfer between the donor and acceptor was calculated as Δ(F*max*/Red), where F*max* is the maximum of emission intensity of Alexa-594 at excitation of Alexa-488; Red is emission maximum of Alexa-594 when excited, Δ indicates a change in attitude Fmax to Red during protein binding to DNA. The highest value of Δ(F*max*/Red) is for the G/T-duplex (0.13), the next is for the A/C-DNA (0.02), the least is for the canonical DNA duplexes (0.01). The Δ(F*max*/Red) values for A/Tg-DNA (0.01) and G/Tg-DNA (0.01) are similar to the canonical DNA duplexes (Table 3). Thus, the Tg containing DNA is not kinked by MutS in contrast to the G/T-duplex.

### Positioning of MutS Bound to Thymidine Glycol Containing DNA

**Figure 5:**
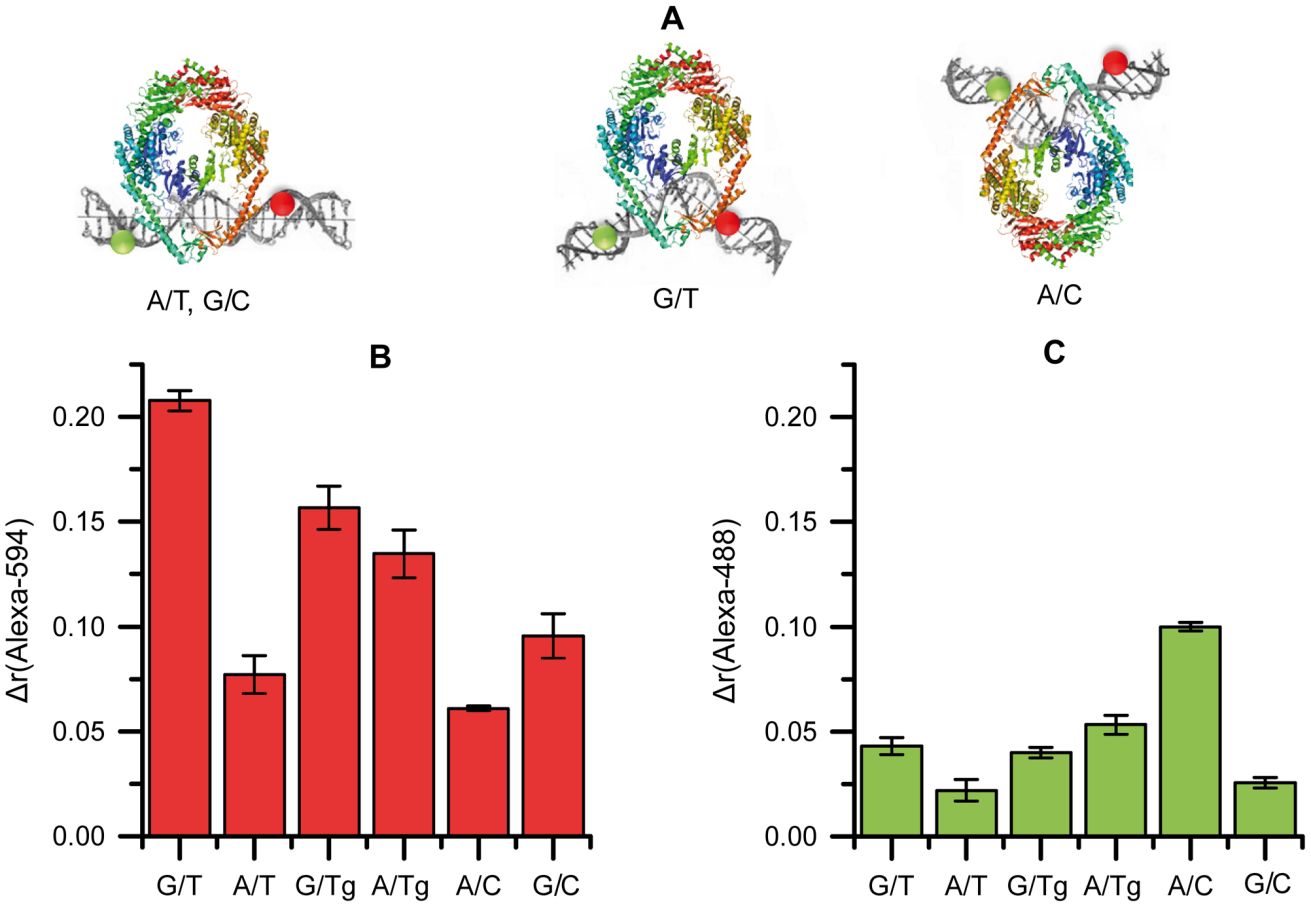
The MutS interaction with DNA duplexes containing FRET pair. **A**, Models of MutS localization on DNA relative to fluorophores Alexa-594 (red) and Alexa-488 (green). The central nucleoside pair is indicated under the cartoons. MutS subunit A which interacts with the mismatch specifically is shown in blue-green; subunit B which forms only non-specific contacts with DNA is shown in yellow-red. B and C, Change in fluorescence anisotropy (Δr) upon maximal binding extent of MutS (total concentration per monomer 125 nM) to DNA (20 nM) containing various central nucleotide pairs: B, for Alexa-488; C, for Alexa-594. Error bars are standard deviations of the mean.

In the specific MutS-DNA complex the amino acid residues of both MutS subunits interact with DNA. However, this binding is asymmetrical – each subunit forms numerous contacts, but they are quite different [50]. Most of these contacts are electrostatic and do not depend on the nucleotide sequence. Due to this fact, MutS can function in different nucleotide context. Only the amino acids of the subunit A (motif Phe-X-Glu) form specific contacts with a mismatch [51]. The MutS location on DNA can be different and depends on the type of the mismatch (Figure 1, step *4*). For example, in the case of G/T pair, MutS forms specific contacts with the mismatched T. This interaction provides a characteristic DNA kink (Figure 5A). In the case of A/C pair, the conserved Phe of MutS contacts the mismatched A forming the kink in the direction opposite to that in the case of G/T pair [50].

The DNA containing two fluorophores at either side of the mismatch can be used as a reporter to evaluate the positioning of MutS bound to a mismatch [35]. Since the environment of the fluorophore can influence the mobility of the dye, measurement of the equilibrium fluorescence anisotropy can be detected if MutS is close to the fluorophores. The closer MutS is to the fluorophore, the greater it restricts the mobility of the fluorophore, resulting in higher anisotropy values.

The fluorescently labeled DNA duplexes V–X were used to determine in which orientation MutS binds the DNA containing Tg (Figure 1, step *4*, b; Figure 2; Figure 5). For both Alexa-594 and Alexa-488 in DNA duplexes V–X, anisotropy values were measured in the absence and in the presence of MutS. In the case of the maximal binding of MutS with the duplexes V–X (Figure 5B), the highest value (0.21) of Δ*r*(Alexa-594) was obtained for G/T-duplex and the lowest – for the canonical DNA (0.075 and 0.090 for the A/T- and G/C-duplexes, respectively) and for the A/C-duplex (0.060). For the duplexes with Tg, interim values (0.13 and 0.15 for the A/Tg- and G/Tg-duplexes, respectively) were observed. For Alexa-488 (Figure 5C), the tendency was different: the maximum value of Δ*r*(Alexa-488) for the A/C-duplex was 0.10, and the values for all the other duplexes were much lower (0.02-0.05). The results can be accounted according to [35]. When kinking the duplex IX with G/T pair, MutS moves closer to Alexa-594 (the distance is less than 5 Å), but is not much closer to Alexa-488. As a result, high anisotropy values are observed for Alexa-594 but not for Alexa-488. The complex of MutS with A/C-DNA (duplex VI) has opposite kink of DNA and due to this fact the opposite tendency of anisotropy values are observed. The values are high for Alexa-488 and low for Alexa-594 (Figures 5B, 5C). In the case of the canonical DNA, MutS has no preferential orientation on the DNA (Figure 5A). The amplitude of anisotropy for free DNA and DNA bound by MutS is minimal. As seen from Figure 5B, in the case of G/Tg and A/Tg containing DNAs, the higher anisotropy values for Alexa-594 are observed in comparison with canonical DNAs and A/C mismatch containing duplex. This effect can indicate that MutS interaction with DNA containing thymidine glycol takes place preferentially in the same manner as with DNA containing G/T mismatch, e.g. the binding occurs from the side of Tg. One can assume that Tg-DNA is kinked in the complex with MutS, as indicated by increasing value of Alexa-488 anisotropy (Figure 5C). However, the degree of bending does not reach 60°.

### Influence of Tg containing DNA ligands on the exchange of ADP in ATPase domains MutS

Specific binding of mismatched DNA with MutS results in the replacement of ADP for ATP in the ATPase domains of the protein (Figure 1, step *5*, case **a**). Activated state of MutS – “sliding clamp” is formed that interacts with the protein MutL, coordinating further stages of MMR [25]. It was necessary to estimate the influence of Tg incorporation into DNA on the rate of nucleotide exchange in ATPase domain of MutS (Figure 1, step *5*, case **b**). In the absence of DNA, MutS can also exchange ADP for a molecule of ATP. However, the presence of bound mismatch containing DNA significantly accelerates the rate of nucleotide exchange [37]. A comparative analysis of nucleotide exchange in the presence of duplexes I–IV and in the absence of DNA was made. The latter approach was reported in [37]. The complex of MutS with a fluorescent analogue of ADP, mant-ADP (2′/3′-O-(N-methyl-anthraniloyl)adenosine-5′-diphosphate), was formed at the first step. As previously shown, the fluorescent group in mant-ADP did not alter biochemical properties of ADP itself and did not influence the binding by proteins [52]. Instead of ATP, which is hydrolyzed by MutS, excess of ADP was used as a competitor for monitoring nucleotide exchange. Dissociation of mant-ADP from the complex with MutS occurs according to the following scheme:
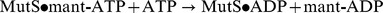



In our experiments, at the point of binding of mant-ADP by MutS the intensity of fluorescence doubled. The excess of ADP was added to the mixture, resulting in an exponential decrease of the signal, the latter reaching initial values (i.e. before adding MutS) (Figure S6 in File S1).

Displacement of mant-ADP from its complex with MutS by specific competitor (ADP) can be characterized by the dissociation rate constant (*k*_off_^mant-ADP^) [37], [38]. It can be regarded as the release rate of ADP from ATPase domain MutS (Table 2, third column).

For the G/T containing duplex II the value of *k*_off_^mant-ADP^ was almost 23 times as high as that in the absence of DNA, which is consistent with the published data [37]. For the canonical and Tg containing duplexes (I, III and IV) a low rate of nucleotide exchange was observed, *k*_off_^mant-ADP^ was within the range of 0.025–0.037 s^−1^. Thus, it was shown that DNAs containing G/Tg and A/Tg pairs do not stimulate the nucleotide exchange in MutS ATPase domains, in contrast to the DNA containing a G/T pair. This may also be the reason for the lack of Tg-DNA repair.

## Conclusions

Our results show that MMR is unlikely to repair Tg containing DNA (Figure 1, step *i*, **b**; Figure 3). The reason could be due to the impossibility of specific contacts between MutS and the Tg residue. According to the published data, the disruption of stacking in DNA double helix along with local structural changes in DNA is important for the MMR system to recognize lesions [42], [53]. In the case of Tg-DNA, we observed double helix destabilization (Figure 1, step *0*, **b**; Table 1). Most likely, it occurred due to the disruption of stacking between thymine glycol and the neighbor bases since the oxidized thymine loses both its aromaticity and planarity. Therefore, the G/Tg and A/Tg pairs could serve as signals for MutS recognition. The Tg residue in DNA has no influence on the rate of ATP hydrolysis by MutS (Figure 1, step *1*, **b**; Table 2) and does not prevent MutS switching to the “turned-on” state (able to bind DNA) [44]. On the other hand, MutS has 3- to 6-fold lower affinity to the Tg containing double stranded DNAs than to the G/T duplex (Figure 1, step *2*, **b**; Table 2). The *K*_d_ values of the MutS complexes with oxidized DNA are insignificantly higher (G/Tg-DNA) or even lower (A/Tg-DNA) than that of the MutS complex with the canonical DNA. Based on our data, one could suggest that the initial MutS interaction with the DNA is realized. The next step is formation of the MutS specific complex with the mismatched DNA which leads to a DNA kink (Figure 1, step *3*, **a**). Such a complex is observed in all crystal structures of MutS with mismatched DNAs [50]. At this step, stacking between the mismatch and the conserved Phe36 is required. MutS does not kink Tg containing DNAs (Figure 4), as Tg cannot be in stacking with Phe. Thus, the initial formation of MutS specific complex with Tg bearing DNA is unlikely, despite the fact that preferential MutS orientation on the Tg containing duplex is similar to its orientation in the case of G/T-DNA (Figure 1, step *4*, case **b**; Figure 5A). Moreover, the nucleotide exchange experiments show that the DNAs with G/Tg and A/Tg pairs do not stimulate the nucleotide exchange in the ATPase domain of MutS. Thus, the MutS transformation to the active “sliding clamp” interacting with MutL is problematic (Figure 1, step *5*, **b**; Table 2). One could suppose that a mismatch could be recognized in the G/Tg or A/Tg containing DNA via Phe36 interaction with the undamaged base of the pair (guanine or adenine). However, it requires the changing of MutS homodimer orientation on the DNA, which is not observed in our experiments. This fact suggests that MutS orientation on the mismatch is chosen before its specific interaction with the noncanonical base pair. Only one MutS subunit is involved in such an interaction, and the protein orientation on DNA cannot be changed.

## Supporting Information

File S1 **Supporting figures.** Figure S1, Analysis of formation of DNA duplex IX containing G/T pair. See Figure 2 and section Materials and Methods for details. Lane 1 – control 45-mer oligonucleotide with Alexa-594, lane 2 – control 13-mer oligonucleotide with Alexa-488. Lanes 3 and 4 – formation of DNA duplex in the absence (lane 3) or in the presence of 5 mM MgCl_2_ (lane 4). Electrophoresis performed in non-denaturing conditions in 20% PAG. Figure S2, Analysis of plasmid DNAs containing the investigated nucleotide pair by electrophoresis 1% agarose gel with ethidium bromide. (gel lanes 3–6, the nucleotide pair indicated over the lanes) Lane 1 – control covalently closed circular DNA (ccc) – plasmid pUC-MMR. Lane 2 – relaxed circular (rc) plasmid DNA obtained from ccc plasmid pUC-MMR by nicking endonuclease Bpu10I treatment. M – DNA ladder. Figure S3, ATP hydrolysis by MutS protein. A, Electrophoresis in 20% PAG with 7 M urea: lane 1 – initial ATP and [γ-^32^P]ATP mixture; lane 2 – products of ATP and [γ-^32^P]ATP hydrolysis by MutS. B, The concentration of phosphate formed by hydrolysis of ATP/[γ-^32^P]ATP (total mixture concentration – 2 µM) by MutS (200 nM of MutS per monomer) in the presence of duplex I (200 nM) at different time points. Standard deviations of the means obtained in three independent experiments are shown in the graph. Figure S4, Analysis of complex formation of MutS with DNA duplex I. Active concentration of MutS per monomer is 31 nM. A, Autoradiography of 4% PAG under non-denaturing conditions (EMSA). Duplex concentrations are indicated over gel lanes, c - initial duplex (5 nM). B, The Scatchard plot for complex of MutS with duplex I. Figure S5, Change in fluorescence anisotropy of the donor (Δ*r*(D), Alexa-488), or the acceptor (Δ*r*(A), Alexa-594) upon binding of MutS to the G/T-duplex IX (20 nM) on total MutS concentration (per monomer). The changes of anisotropy values in the process of MutS binding with DNA were obtained by subtracting the initial value for the duplex alone. Anisotropy is given without background signals. *K*_d_ for complex MutS with G/T-DNA IX is 26±6 nM measured by Alexa-594 anisotropy change during the MutS binding. Standard deviations of the means obtained in three independent experiments are shown on the curves. Figure S6, The time dependence of mant-ADP fluorescence intensity (*I*) in experiment on ADP exchange by MutS in the presence of DNA duplex I. Reaction mixture: *1* – with mant-ADP but without MutS, *2* – after addition of MutS, *3* – after addition of excess of unlabeled ADP to complex of MutS with mant-ADP.
(DOC)
