# Correction: Is Thymidine Glycol Containing DNA a Substrate of *E. coli* DNA Mismatch Repair System?

**DOI:** 10.1371/journal.pone.0118035

**Published:** 2015-02-10

**Authors:** The PLOS ONE Staff

There is an error in the last sentence of the fourth paragraph of the Introduction. The correct sentence is: Then an exchange of ADP with ATP in ATP domains of MutS takes place (Figure 1, step *5*, **a**) and there is formed an ultimate recognition complex, which being in the “sliding clamp” conformation initiates an MMR reaction cascade involving MutL and MutH (Figure 1, step *i*, **a**).

There are multiple errors in the structures of the duplexes I–IV (5′→3′/3′→5′) shown in the “Synthetic DNA Fragments” subsection of the Materials and Methods. Please see the correct structures here.

CAAGCCTATGCCCTCAGCACCCA**G**GGTGCC I (G/C)

GTTCGGATACGGGAGTCGTGGGT**C**CCACGG

CAAGCCTATGCCCTCAGCACCCA**G**GGTGCC II (G/T)

GTTCGGATACGGGAGTCGTGGGT**T**CCACGG

CAAGCCTATGCCCTCAGCACCCA**G**-GGTGCC III (G/Tg)

GTTCGGATACGGGAGTCGTGGGT**Tg**CCACGG

CAAGCCTATGCCCTCAGCACCCA**A**-GGTGCC IV (A/Tg)

GTTCGGATACGGGAGTCGTGGGT**Tg**CCACGG
