# Supplementary material for: Is Thymidine Glycol Containing DNA a Substrate of E. coli DNA Mismatch Repair System?
Source: PLoS One. 2014 Aug 18;9(8):e104963. doi: 10.1371/journal.pone.0104963 (PMC4136841; doi:10.1371/journal.pone.0104963)
Supplement: File S1 — Supporting figures. Figure S1, Analysis of formation of DNA duplex IX containing G/T pair. See Figure 2 and section Materials and Methods for details. Lane 1 – control 45-mer oligonucleotide with Alexa-594, lane 2 – control 13-mer oligonucleotide with Alexa-488. Lanes 3 and 4 – formation of DNA duplex in the absence (lane 3) or in the presence of 5 mM MgCl2 (lane 4). Electrophoresis performed in non-denaturing conditions in 20% PAG. Figure S2, Analysis of plasmid DNAs containing the investigated nucleotide pair by electrophoresis 1% agarose gel with ethidium bromide. (gel lanes 3–6, the nucleotide pair indicated over the lanes) Lane 1 – control covalently closed circular DNA (ccc) – plasmid pUC-MMR. Lane 2 – relaxed circular (rc) plasmid DNA obtained from ccc plasmid pUC-MMR by nicking endonuclease Bpu10I treatment. M – DNA ladder. Figure S3, ATP hydrolysis by MutS protein. A, Electrophoresis in 20% PAG with 7 M urea: lane 1 – initial ATP and [γ-32P]ATP mixture; lane 2 – products of ATP and [γ-32P]ATP hydrolysis by MutS. B, The concentration of phosphate formed by hydrolysis of ATP/[γ-32P]ATP (total mixture concentration – 2 µM) by MutS (200 nM of MutS per monomer) in the presence of duplex I (200 nM) at different time points. Standard deviations of the means obtained in three independent experiments are shown in the graph. Figure S4, Analysis of complex formation of MutS with DNA duplex I. Active concentration of MutS per monomer is 31 nM. A, Autoradiography of 4% PAG under non-denaturing conditions (EMSA). Duplex concentrations are indicated over gel lanes, c - initial duplex (5 nM). B, The Scatchard plot for complex of MutS with duplex I. Figure S5, Change in fluorescence anisotropy of the donor (Δr(D), Alexa-488), or the acceptor (Δr(A), Alexa-594) upon binding of MutS to the G/T-duplex IX (20 nM) on total MutS concentration (per monomer). The changes of anisotropy values in the process of MutS binding with DNA were obtained by subtracting the initial value for [file pone.0104963.s001.doc]

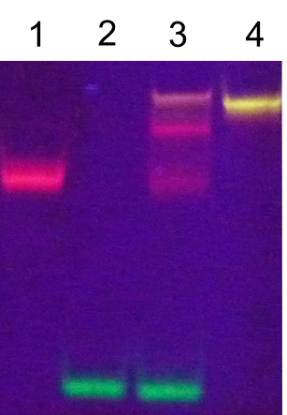


**Figure S1. Analysis of formation of DNA duplex IX containing G/T pair.** See Figure 2 and section Materials and Methods for details. Lane 1 – control 45-mer oligonucleotide with Alexa-594, lane 2 – control 13-mer oligonucleotide with Alexa-488. Lanes 3 and 4 – formation of DNA duplex in the absence (lane 3) or in the presence of 5 mM MgCl_2_ (lane 4). Electrophoresis performed in non-denaturing conditions in 20% PAG.

**
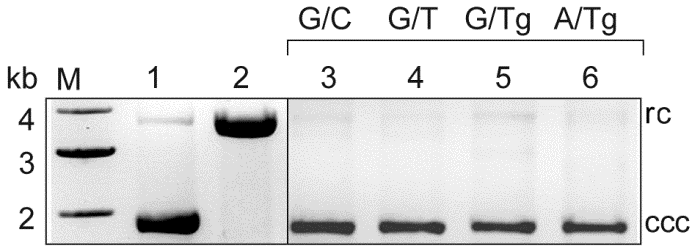
**

**Figure S2.** **Аnalysis of plasmid DNAs containing the investigated nucleotide pair by electrophoresis** **1% agarose gel with ethidium bromide** (gel lanes 3-6, the nucleotide pair indicated over the lanes). Lane 1 – control covalently closed circular DNA (ccc) – plasmid pUC-MMR. Lane 2 – relaxed circular (rc) plasmid DNA obtained from ccc plasmid pUC-MMR by nicking endonuclease Bpu10I treatment. М – DNA ladder.


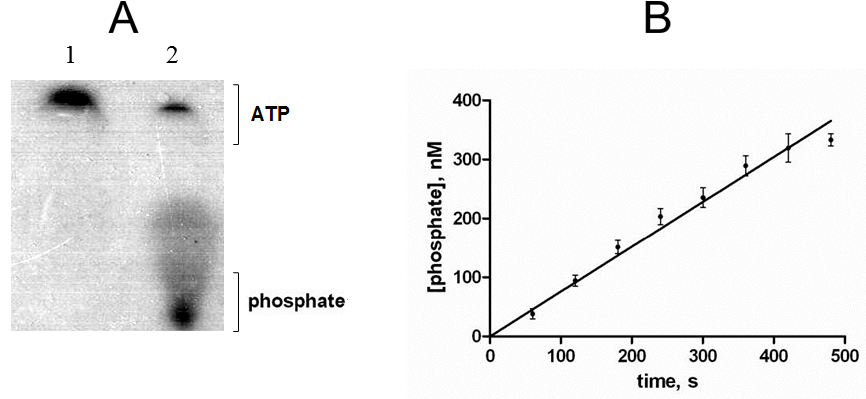


**Figure S3.** **ATP hydrolysis by MutS protein. A,** Electrophoresis in 20% PAG with 7 M urea: lane 1 – initial ATP and [γ-^32^P]ATP mixture; lane 2 – products of ATP and [γ-^32^P]ATP hydrolysis by MutS. **B,** The concentration of phosphate formed by hydrolysis of ATP/[γ-^32^P]ATP (total mixture concentration – 2 μM) by MutS (200 nM of MutS per monomer) in the presence of duplex I (200 nM) at different time points. Standard deviations of the means obtained in three independent experiments are shown in the graph.


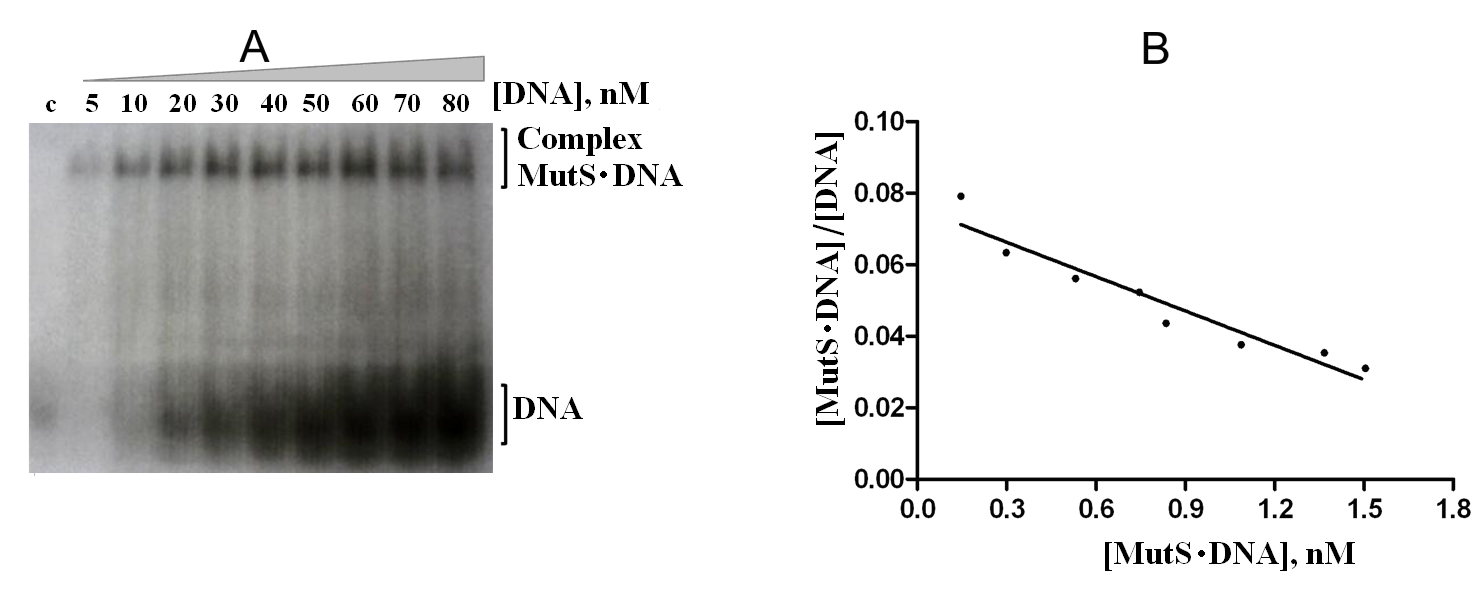


**Figure S4.** **Analysis of complex formation of MutS with DNA duplex I.** Active concentration of MutS per monomer is 31 nM. **A,** Autoradiography of 4% PAG under nondenaturing conditions (EMSA). Duplex concentrations are indicated over gel lanes, c - initial duplex (5 nM). **B,** The Scatchard plot for complex of MutS with duplex I.


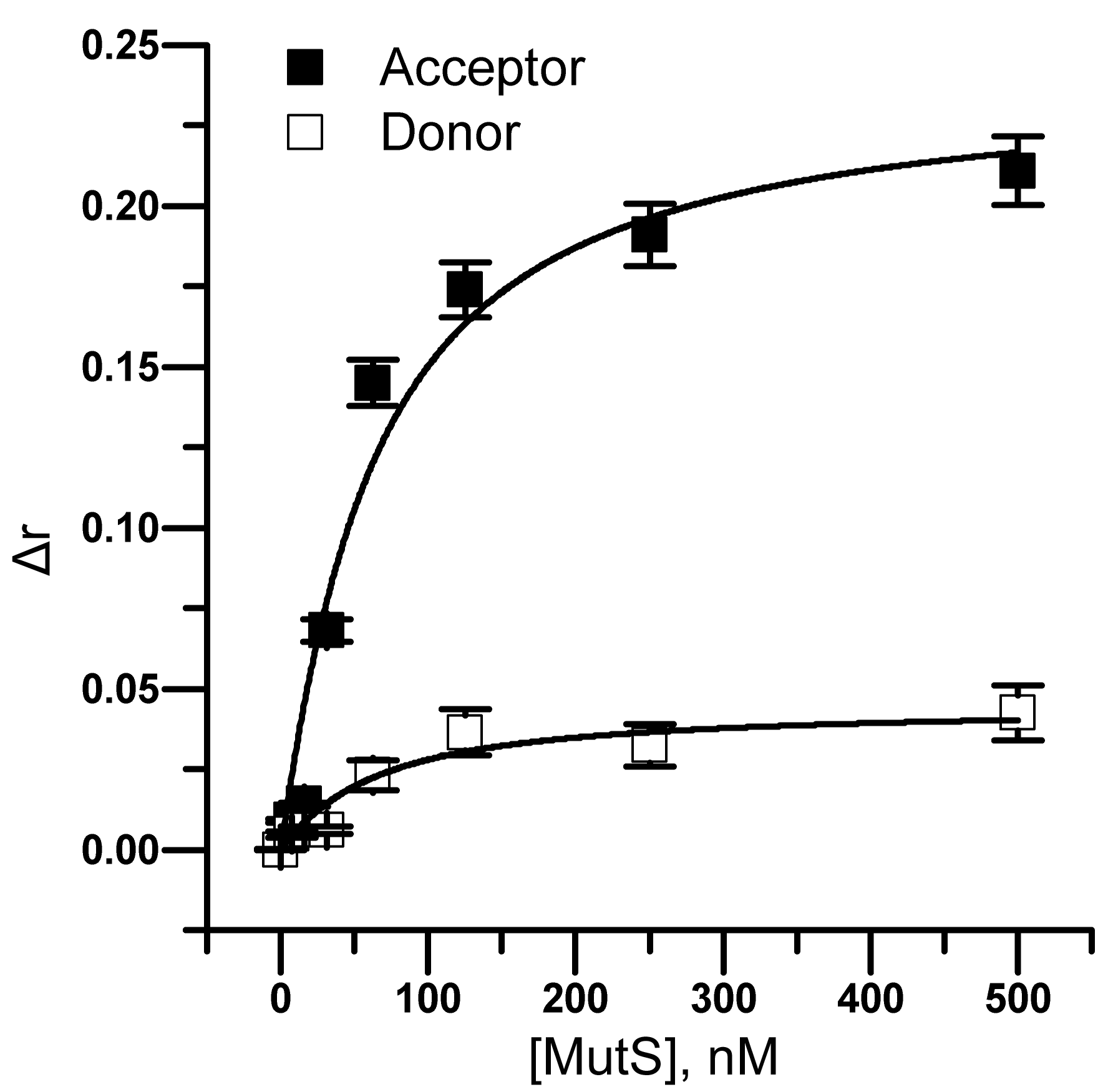


**Figure S5.** **Change in fluorescence anisotropy of the donor (∆*r*(D), Alexa-488), or the acceptor (∆*r*(A), Alexa-594) upon binding of MutS to the G/T-duplex IX (20 nM) on total MutS concentration (per monomer).** The changes of anisotropy values in the process of MutS binding with DNA were obtained by subtracting the initial value for the duplex alone. Anisotropy is given without background signals. *K*_d_ for complex MutS with G/T-DNA IX is 26±6 nM measured by Alexa-594 anisotropy change during the MutS binding. Standard deviations of the means obtained in three independent experiments are shown on the curves.


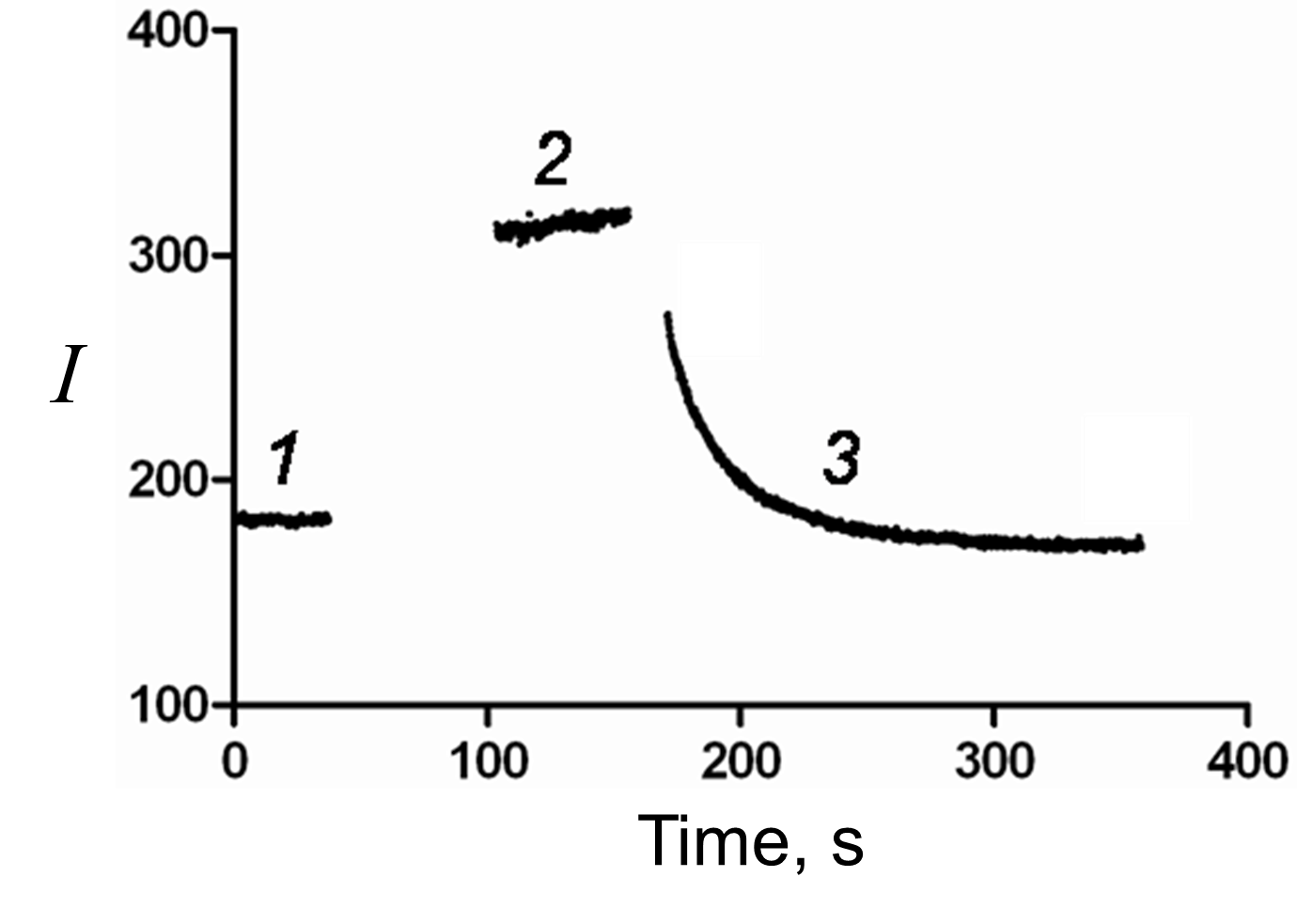


**Figure S6.** **The time dependence of mant-ADP fluorescence intensity (*I*) in experiment on ADP exchange by MutS in the presence of DNA duplex I.** Reaction mixture: ***1*** – with mant-ADP but without MutS, ***2*** – after addition of MutS, ***3*** – after addition of excess of unlabeled ADP to complex of MutS with mant-ADP.
